# Application of Machine Learning in Ethical Design of Autonomous Driving Crash Algorithms

**DOI:** 10.1155/2022/2938011

**Published:** 2022-09-24

**Authors:** Yineng Xiao

**Affiliations:** Advanced Institute of Information Technology, Peking University, Hangzhou 311200, China

## Abstract

The age of algorithms is here, and it is really changing people's lives. More and more ethical problems related to algorithms have attracted people's attention, but the related ethical research is still far behind the research of algorithms. As more intelligent algorithms emerge in an endless stream, there will also be a lot of algorithmic ethical issues. On the other hand, with the continuous improvement of the development level of the automobile industry, people have a stronger demand for the safety and stability of modern transportation, and more and more autonomous driving technology has been promoted and applied in the market. At present, most of the studies on the longitudinal collision avoidance system of vehicles use collision warning or emergency braking to avoid collision. However, when the vehicle is in a special situation such as high speed and slippery road, emergency steering is more effective. In order to further improve the vehicle safety and ethical algorithm design points, this article revolves around vehicle lateral active collision avoidance control method research, the collision avoidance decision-making, and path planning and collision avoidance transverse vehicle longitudinal motion control is analyzed, and based on automated driving simulation experiment, the tests carried out to verify the designed control strategy. The experimental results show that the proposed method not only has a good effect of preventing automatic driving collision but also can meet the requirements of algorithm ethics. This research can effectively guide the research of algorithmic ethics in the field of autonomous driving and effectively reduce the occurrence of traffic accidents.

## 1. Introduction

With the development of data collection and storage technology, big data scenarios can be seen everywhere in our life. The key to the big data era is not just that the amount of data is exploding exponentially, but that machine learning algorithms can dramatically improve the ability to collect and analyze data [1, 2]. This allows data analysis to go beyond traditional statistical sampling to the correlation of different datasets, making predictions about the whole or different individuals and influencing people's decisions. Therefore, the importance of machine learning algorithms is self-evident, and some people even call the era of big data the era of intelligent algorithms. The rapid development of information technology brings convenience to society but also brings us many technical and ethical problems. This requires that ethical research on machine learning should be prioritized, interdisciplinary research should be carried out, forward-looking and constraining guidance should be strengthened, risks should be minimized, and the safe, reliable, and controllable development of machine learning algorithms should be ensured [3, 4].

The intelligent algorithm has become the engine of the rapid development of society. The core of technology development is mainly reflected in the decision-making of the algorithm. How to make the algorithm have moral judgment in the generation and design is very important. This requires artificial intelligence algorithms to be ethically acceptable algorithms or ethical algorithms and to make the decision-making of autonomous systems highly reliable and secure. In this sense, the moral algorithm is a basic principle and the bottom line is to realize the functional security of artificial intelligence. Algorithmic ethics, as an extension of technical ethics, is completely different from technical ethics in this respect [5, 6]. Similar differences are not only unique to algorithmic ethics but also common in new age new things ethics. It is a great challenge in the era of big data how to apply traditional ethical concepts to algorithm ethics and then establish algorithmic ethical norms and avoid algorithmic ethics from getting out of control, which is also worthy of extensive research. By extension, the research and analysis experience of algorithmic ethics can also be used for reference in other new ethical fields. Strengthening the research of algorithmic ethics is also promoting the continuous improvement and development of ethical theories in the new era.

With the continuous breakthrough of artificial intelligence technology, the continuous development of scientific and technological forces is accelerating the transformation of the entire automobile travel industry. Intelligent driving has become one of the main development directions of the automobile industry imperceptibly. However, as the vehicle drives more and more with high degree of automation, different countries' different scholars began to research for the development of intelligent car gradually in the real road test scenarios, and the smart cars in the road test process also exposed the limitations in many fields, and even some test scenarios created serious mistakes, which led to the inevitable accidents [7, 8]. According to the statistics of the World Health Organization (WHO), the deaths caused by traffic safety problems have become one of the major causes of death in the world population every year. In the current traffic environment, a lot of traffic accidents and casualties have been caused by unreasonable urban traffic planning, poor road conditions, dangerous driving behaviors, and weak traffic safety awareness. How to improve traffic safety reasonably and effectively has always been a thorny worldwide problem and therefore has become a very hot research field in academia and industry. The current safety research in the traffic environment mainly focuses on how to avoid collisions between vehicles and vulnerable road users such as pedestrians. Among them, the problem of anticollision between vehicles has been paid attention to earlier by academians and industry. At present, the research of intervehicle collision warning algorithms is mainly divided into perceptual experience-based algorithms [9, 10].

**Figure 1:**
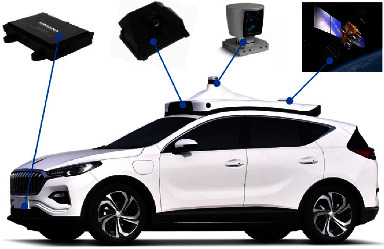
structure of the intelligent vehicle environment sensing system.

Vehicle collision and rear-end collision are the most common traffic accidents. Vehicle collision often affects a wide range and causes a high probability of injury to vehicles and occupants. The researchers analyzed rear-end collisions and found that about 80 percent of the accidents were caused by drivers not responding quickly enough, and more than half of the accidents were caused by rear-end collisions. German researchers have analyzed the results of traffic accidents and found that most accidents can be avoided if drivers can anticipate a possible traffic safety problem a second in advance and take the right evasive action in time. Therefore, the study of vehicle active collision avoidance method is an important direction to avoid the increase of traffic accidents, ensure the efficiency of traffic system, and maintain traffic safety. With an increase in car ownership, the situation of road traffic safety becomes more and more serious. The rapid development of intelligent vehicles, such as autonomous vehicles, intelligent connected vehicles (ICVs), and vehicles equipped with advanced driver assistance systems (ADAS), is of great significance to reduce the occurrence and injury of traffic accidents. Studies have shown that smart car technology will reduce traffic accidents by 50 to 80 percent and improve traffic efficiency by 10 to 30 percent. Smart cars mainly use on-board sensors such as, cameras, liDAR, and intelligent network communication devices to sense the surrounding environment, as shown in Figure 1. Among them, the camera sensor algorithm has strong adaptability, high optical measurement accuracy, and low cost so that the camera sensor can obtain more abundant data information. According to the different environment sensing systems, they can be divided into sensing technology, communication technology, and fusion technology. Among them, traffic conflict research of perception technology mainly includes three categories: computer vision , radar(LIDAR, millimeter wave radar, and ultrasonic radar), and multisensor fusion. Machine learning technology has great advantages in environmental sensing [11, 12]. Perception-communication fusion is the mainstream method to solve traffic conflicts. For intelligent vehicles, it is necessary to solve environmental data fusion, vehicle trajectory tracking, active collision avoidance, high level of positioning accuracy and communication accuracy, and other technical problems. Machine learning technology has been widely used in the field of autonomous driving.

Traditional vehicle drivers need to deal with a lot of driving in the process of moving information and making the right decisions and have the effect of regulation and control; the driver while in the transportation system plays the main role. However, individuals are highly random and cannot be predicted directly. Safety, especially the active safety, is the main factor that promotes the growth of automatic driving vehicle demand, and self-driving vehicles can be very good to avoid traffic accidents caused by drivers because it is based on a highly integrated computer technology among many other emerging science and technology developments in the integration of smart car which is AI technology and an inevitable result of the rapid development of computer intelligent control technology [13, 14]. It has a broad application prospect in the field of national defense and national economy. The development of automatic driving technology plays a positive role in improving road traffic safety performance. Before the early development of emergency collision avoidance systems to improve driving safety, the industry commonly used passive safety control systems, including the antilock brake system (ABS), traction control system (TCS), and electronic stability controller (ESC). While these passive safety systems are important in reducing the severity of vehicle crashes and, in certain circumstances, can help the driver avoid them, even if these safety systems are fully operational, the driver will still be in charge of the vehicle during periods when the vehicle is in danger. The next step of automobile safety technology is mainly to develop active safety technology represented by the advanced driving assistance system (ADAS). When a vehicle encounters a dangerous situation for collision avoidance, the active collision avoidance movement can be divided into two categories, namely, changing the longitudinal motion state of the vehicle through emergency braking for collision avoidance and steering or braking coordinated steering to make the vehicle bypass obstacles in the form of emergency lane change for collision avoidance [15, 16].

Similarly, intelligent vehicles represented by autonomous vehicles will face more severe challenges in complex traffic scenarios with the improvement of the level of autonomous driving. At present, under the challenge of complex traffic scenes, self-driving car accidents occur frequently. However, from the perspective of traffic system, we still face the following problems: (1) It is a long-term process for autonomous driving to replace traditional driving, and it has not been decided when the replacement will be completed. In the future, it will still be dominated by traditional driving vehicles, supplemented by autonomous driving, but the proportion of autonomous driving is increasing. (2) The driving behavior characteristics of autonomous driving and traditional driving vehicles are quite different, and the traffic safety risk situation under the mixed driving condition is not clear and may even be lower than that under the full traditional driving condition at present [17, 18]. The inflection point and situation is unclear that whether the trend of traffic safety risk will continue to improve or turn better after deterioration. Based on the above background, this paper focuses on the construction of collision warning and intelligent vehicle test scenarios for autonomous driving to improve the driving safety of intelligent vehicles in complex vehicle traffic environments to study the application of machine learning in the ethical design of autonomous driving crash algorithms.

## 2. Related Work

In recent years, technology for good deeds has increasingly become the forefront of ethics and the mainstream value of various organizations, including technology companies. In this sense, science and technology are no longer regarded as abstract knowledge and capabilities but are committed to penetrating the power of science and technology into the places where society needs it to improve human well-being. The artificial intelligence and machine deep learning algorithms make artificial intelligence to be widely used in the social field. This is a scientific and technological innovation with unknown results but also a far-reaching social ethical experiment in the history of human civilization [19, 20]. It mainly focuses on the following issues: The first issue is the reflection of algorithm ethics. The second one is the research of algorithm design ethics. In terms of artificial intelligence as technology, artificial intelligence is designed to achieve its function. The third one is the value load of the algorithm. Technology and algorithm are both input and output processes in nature. The value load of an algorithm is positive and negative. If the value is neutral, there is no need to discuss it. The fourth issue is the moral decision of algorithms. Excellent algorithms can accurately and quickly complete the set program tasks. To judge the excellence of algorithms, we should not only take the shared core value of human beings as the standard but also respect the reasonable value demands of different clusters. Morality is not a simple judgment of right or wrong but refers to how the good behavior and decision of the subject affect others in the social environment so that the behavior and decision of others conform to the mainstream norms of behavior [21, 22]. How to make the algorithm decision moral is mainly reflected in the moral decision of the algorithm.

Reducing traffic accidents is a major public safety challenge the world is facing today. Traffic accident risk analysis is very important for optimizing public transport, creating safer routes, and improving the cost effectiveness of transport infrastructure, all with the common aim of making driving safer. In view of its importance, accident analysis has been the subject of many researches in the past decades, and a large number of researchers at home and abroad have been studying accident analysis and other related fields tirelessly [23, 24]. The analysis of the causes of traffic accidents and the exploration of the potential influencing factors of complex-driving environment elements on the risk of accidents can give theoretical support to the design of intelligent driving safety system and improve the safety of intelligent driving. According to different accident analysis algorithms, it can be roughly divided into two methods: traditional machine learning method and neural network method. The risk analysis algorithm based on the traditional machine learning method is first through the characteristics of engineering or other data preprocessing method characteristics of complex native driving environmental data processing and feature extraction, and then the machine learning algorithm is adopted to building environment characteristics and the relationship between the accident risk driving and the accident risk model and based on the model of information mining. Among them, decision tree and AdaBoost are widely used classification regression algorithms with good performance in traditional machine learning [25, 26].

Risk analysis methods based on neural networks usually use different types of neural networks to analyze the relationship between accident risk and driving environment. Such methods often use the powerful feature extraction and mapping simulation capabilities of neural networks to achieve efficient and accurate information extraction. However, due to the poor interpretability of neural networks, it is generally impossible to quantitatively analyze the relationship between driving environment elements and the severity of accidents, so it is often used to predict the accident risk based on driving environment elements [27, 28]. In the field of intelligent driving, the rapid development of artificial intelligence technology has greatly strengthened the perception and decision-making ability of intelligent cars to the surrounding driving environment. The intelligent driving system contains a lot of perception modules based on neural networks. Under ideal conditions, we hope to get the correct results for the output values of the intelligent vehicle perception system in different environments. However, it is essentially a probability problem. The current deep learning algorithm can only give a specific result for an output but cannot give how high the model's confidence is for the whole output result. This problem leads to that in the practical application process, and deep neural network can give a particularly good result in most cases, but occasionally gives a particularly bad result in extreme cases. However, it is the particularly bad result that usually leads to the occurrence of traffic accidents in the end [29, 30].

Internet of vehicles (IoV), also called V2X communication (vehicle-to-everything), is an emerging communication technology for transportation systems. It aims to establish communication between vehicles and their surrounding environment and provide vehicles with comprehensive and complete road traffic information. For the back-end algorithm, strategy processing is done so as to achieve a more intelligent and efficient traffic effect. V2X communications include a variety of communications, e.g., vehicle-to-vehicle (V2V) communication, vehicle-to-infrastructure (V2I) communication between the vehicle and surrounding facilities, and vehicle-to-pedestrian communication (V2P), and vehicle-to-network (V2N) communication. For future autonomous driving and intelligent transportation scenarios, the Internet of vehicles can be said to be an indispensable means of information perception. At present, the mainstream automatic driving adopts the on-board sensor system to realize the information sensing function. With the rapid development of deep learning, image processing, and other fields, it has become a mainstream solution to obtain the surrounding environment information through the on-board camera and radar system. However, these sensors all have the same limitation; that is, they can only perceive the information within the line-of-sight range, and in addition to the limited coverage, they are unable to do anything about the scene with occlusion. By establishing a communication connection between the vehicle and the surrounding environment, the required information is transmitted to each other, so as to realize the purpose of sensing, which will be a good solution to the problems faced by these sensor systems [31, 32].

**Figure 2:**
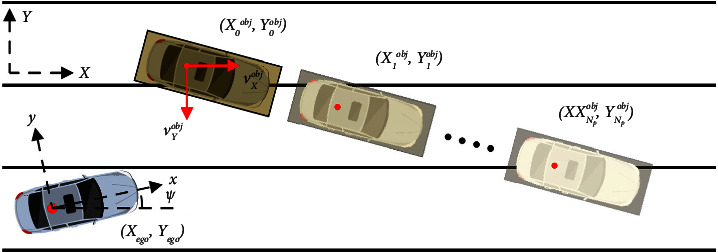
Schematic diagram of automatic driving obstacle intelligent recognition.

The research on autonomous driving technology started earlier in foreign countries. The first self-driving project was led by Carnegie Mellon University in the United States in 1995. That same year, the National Autonomous Highway System Alliance was formed to support autonomous driving programs. Developed countries such as Europe and Japan have also started research and investment in autonomous driving related fields, which has greatly promoted the progress of intelligent vehicles. Tech companies have also set their sights on autonomous driving. In July 2016, Tesla released the Tesla Model 3, and upgraded the Model 3 in June 2018. The new version has a field of view of 360° and a detection distance of up to 250 meters. The Model 3 is equipped with a total of eight cameras around the body, and 12 ultrasonic sensors are added to compensate for the limitations of the vision system. The detection rate is not limited by the material properties of the object, and the sensing distance and accuracy improved greatly. The research on autonomous driving technology is relatively new to China, but it is developing well. In 1992, China's first truly driverless car was born. Domestic universities such as Tsinghua University, National University of Defense Technology, and others are among the first to start research on autonomous driving technology. In recent years, the domestic research on autonomous driving technology has been extended to the entire automobile industry, and the traditional automobile enterprises and the emerging car manufacturing forces and Internet enterprises have invested a lot of manpower and financial resources in the research of autonomous driving related technology. With the iteration of intelligent Internet technology, the wave of automobile intelligence is also coming into existence, and the attribute of automobile as a simple mobile tool is gradually transformed to the second space as a mobile intelligent terminal. Colleges and universities are developing automated driving technology research in the real car development, and the test cost is too high, but the use of smart car as an alternative to the intelligent vehicle is reasonable, and its price is lower and simple lines, control, and adjustment easier manipulation could be seen in the application of automated driving technology, and the intelligent car platform programming work can be reduced a lot. The research focus can be more on the design and implementation of the algorithm itself. Before the vehicle driving area is established, the motion trajectories of moving obstacles in front of the vehicle need to be predicted. A prediction model for moving obstacles is established based on the motion state of autonomous vehicles, relative position, and relative speed of moving obstacles, as shown in Figure 2. Since this figure is only a simple explanation of the intelligent recognition principle of automatic driving obstacles, the formula in it is only an example, so no specific explanation is given in the paper.

With the continuous development of automobile and related industries and the frequent occurrence of traffic accidents such as collision and rear-end collision, automobile active collision avoidance technology has become the key, and experts are paying more and more attention to the direction of automobile active safety. The vehicle active collision avoidance system detects the surrounding environment, including the relative position and speed of pedestrians and obstacles through sensors such as cameras, lasers, and radar, and judges whether the vehicle is at risk of collision according to the relative distance with obstacles, and warns the driver through images or sounds. If the driver fails to take timely measures to avoid collision after exceeding the safe range, the active collision avoidance system of the vehicle will automatically take over the vehicle to avoid the accident risk. In some cases, vehicles cannot avoid rear-end collision by braking [33, 34]. When vehicles are parallel or overtaking, lateral collision accidents are easy to occur. Through active steering, the risk of lateral collision is reduced by the control strategy and the related algorithm considering whether the lateral distance between two vehicles is within the safe range. A vehicle only by longitudinal emergency braking or laterally changing is unable to avoid collision accident, then the vehicle encounters obstacles ahead, and the active collision avoidance system by the relative position and relative velocity judge whether there is a collision threat or risk control vehicle automatic obstacle avoidance of collision if the vehicle environment to the condition of lane changing is not in conformity. It can control the automatic braking of the vehicle, comprehensively consider the injury degree of the accident, avoid the risk and reduce the collision injury as much as possible [35]. Many companies and researchers in the field of collision avoidance system found that most studies only consider a safe distance, longitudinal or transverse collision, etc., without considering the collision caused by the crew casualties, but in the real scenario, under a lot of emergency cars, collision cannot be avoided when the driver is easy to emotional tension. Unable to rationally and timely make a reasonable judgment, the driver's limited driving experience is unable to deal with this kind of accident; if the driving right is still in the hands of the driver, it is likely to cause the vehicle to lose control, causing serious casualties. If the vehicle can automatically take over at this time, according to a large number of collision simulation data much higher than human's own computing ability, the use of machine learning algorithm to develop the collision avoidance strategy to minimize casualties has a very important practical significance for reducing the injury of road traffic accidents. After that, the main contributions of this paper are given as follows:

1. In this paper, a Kalman filter model is proposed for the first time for ethical design of autonomous driving crash
2. This paper not only has strong theoretical value but also has certain application prospect

## 3. Kalman Filtering Based Ethical Design of Autonomous Driving Crash

### 3.1. Kalman Filter Introduction

The Kalman filter is not similar to the traditional filter in the frequency domain, but a state predictor in the time domain, which eliminates the time domain and frequency domain transformation step. The basic idea of the extended Kalman filter is to linearize the nonlinear system and then carry out the Kalman filter, so it is a pseudo-nonlinear Kalman filter. The Kalman filter is an operation based on recursion, which uses linear minimum variance error to estimate the state sequence of dynamic system effectively. In this way, only the parameters of the current and previous process can be stored, which greatly reduces the system's requirement for data storage and improves the calculation speed. In addition, the mathematical model of Kalman filter is simple, and it has good real-time performance and good fast response, which can track the autonomous vehicle in real time. The following state space model is used to describe the dynamic system:

(1) $$\begin{array}{c} X\left(k+1\right)=AX\left(k\right)+BW\left(k\right)\text{.} \end{array}$$

Then, the error of the hidden layer can be given as follows:

(2) $$\begin{array}{c} Y\left(k\right)=HX\left(k\right)+V\left(k\right)\text{,} \end{array}$$

where *X*(*k*) represents the system state vector at time *k*, *Y* (*k*) is the observation vector of the system, and *A* is the shape transition matrix. The system state estimation at time *k* + 1 is predicted by the state estimation at time *k* as follows:

(3) $$\begin{array}{c} \hat{X}\left(k+1\;|\;k\right)=A\hat{X}\left(k\;|\;k\right)+BU\left(k\right)\text{.} \end{array}$$

The covariance matrix at time *k* + 1 is predicted by the modified covariance matrix at time *k* and the process noise *Q*:

(4) $$\begin{array}{c} P\left(k+1\;|\;k\right)=AP\left(k\;|\;k\right)A^{T}+Q\text{.} \end{array}$$

Find the Kalman gain *K* from the predicted covariance matrix as

(5) $$\begin{array}{c} K\left(k+1\right)=P\left(k+11k\right)H^{T}{\left[HP\left(k+1\;|\;k\right)H^{T}+R\right]}^{-1}\text{.} \end{array}$$

According to the predicted state estimation, observed value, and Kalman gain *K*, the predicted system state value is modified as

(6) $$\begin{array}{c} \hat{X}\left(k+1\right)=\hat{X}\left(k+1\;|\;k\right)+K\left(k+1\right)\left[Y\left(k+1\right)-H\hat{X}\left(k+1\;|\;k\right)\right]\text{.} \end{array}$$

Updating the system covariance matrix at time *k* + 1, we get

(7) $$\begin{array}{c} P\left(k+1\right)=\left[1-K\left(k+1\right)H\right]P\left(k+1\;|\;k\right)\text{,} \end{array}$$

where *k* is the set of real numbers, and *P (k* *+* 1) is belonged to [0, 1].

### 3.2. Automatic Driving Collision Analysis by the Kalman Filter

The error between the predicted position and the actual position of the vehicle target is reduced continuously by solving the recursive equations of the prediction, and the desired goal is achieved. Finally, the vehicle position is predicted as

(8) $$\begin{array}{c} s\left(k+1\right)=s\left(k\right)+v\left(k\right)T+0.5T^{2}a\left(k\right)\text{,} \end{array}$$

(9) $$\begin{array}{c} v\left(k+1\right)=v\left(k\right)+Ta\left(k\right)\text{.} \end{array}$$

Assume that the vehicle state at time *k* is

(10) $$\begin{array}{c} X\left(k\right)=\left[\begin{array}{c} z\left(k\right)\\ x\left(k\right)\\ v_{z}\left(k\right)\\ v_{x}\left(k\right) \end{array}\right]\text{.} \end{array}$$

The acceleration *A* (*k*) is composed of two parts, maneuvering acceleration *U* (*k*) and random acceleration *W* (*k*). The motion state equation of the vehicle is as follows:

(11) $$\begin{array}{c} \left[\begin{array}{c} z\left(k+1\right)\\ x\left(k+1\right)\\ v_{z}\left(k+1\right)\\ v_{x}\left(k+1\right) \end{array}\right]=\left[\begin{array}{cccc} 1 & 0 & T & 0\\ 0 & 1 & 0 & T\\ 0 & 0 & 1 & 0\\ 0 & 0 & 0 & 1 \end{array}\right]\left[\begin{array}{c} z\left(k\right)\\ x\left(k\right)\\ v_{z}\left(k\right)\\ v_{x}\left(k\right) \end{array}\right]+\left[\begin{array}{cc} 0.5T^{2} & 0\\ 0 & 0.5T^{2}\\ T & 0\\ 0 & T \end{array}\right]\left[\begin{array}{c} u\left(k\right)\\ u\left(k\right) \end{array}\right]+\left[\begin{array}{cc} 0.5T^{2} & 0\\ 0 & 0.5T^{2}\\ T & 0\\ 0 & T \end{array}\right]\left[\begin{array}{c} w\left(k\right)\\ w\left(k\right) \end{array}\right]\text{.} \end{array}$$

The vehicle observation equation is as follows:

(12) $$\begin{array}{c} Y\left(k\right)=\left[\begin{array}{cccc} 1 & 0 & 0 & 0\\ 0 & 1 & 0 & 0 \end{array}\right]\left[\begin{array}{c} z\left(k\right)\\ x\left(k\right)\\ v_{z}\left(k\right)\\ v_{x}\left(k\right) \end{array}\right]+v\left(k\right)\text{.} \end{array}$$

Assuming that the vehicle moves at a constant speed in the time interval *T*, the maneuvering acceleration *U* (*k*) can be ignored, and the equation of state of the system is

(13) $$\begin{array}{c} X\left(k+1\right)=\left[\begin{array}{cccc} 1 & 0 & T & 0\\ 0 & 1 & 0 & T\\ 0 & 0 & 1 & 0\\ 0 & 0 & 0 & 1 \end{array}\right]X\left(k\right)+\left[\begin{array}{cc} 0.5T^{2} & 0\\ 0 & 0.5T^{2}\\ T & 0\\ 0 & T \end{array}\right]w_{2\times 1}\left(k\right)\text{.} \end{array}$$

Because some deep school models require a high amount of experimental data and the structure of the model is relatively complex and because the Kalman filter in this paper is a conventional machine school model, the deep school method is not selected in this paper.

### 3.3. The Flow of the Proposed Method

**Figure 3:**
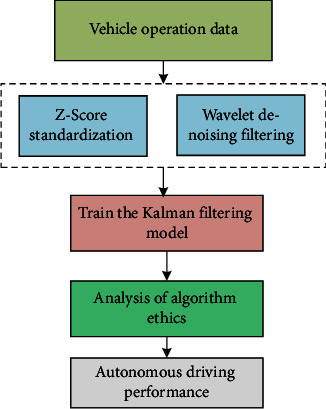
The framework of the proposed method.

Based on the relevant theories of Kalman filter and equations (1)–(13) introduced above, Figure 3 gives the application of machine learning in ethical design of autonomous driving crash algorithms. It is worth noting that this paper also analyzes the ethics of Kalman algorithm and evaluates the performance of autonomous driving, the ethics of the algorithm is analyzed, and the performance evaluation of automatic driving is evaluated.

## 4. Experimental Results and Analysis

### 4.1. Experimental Data Introduction

The China Traffic Accident Investigation System (NAIS) database is a collection of serious traffic accidents. Started at the earliest, China has one of the most serious traffic accident database in detail and it includes eight sites, such as site by university, institute of traffic safety and judicial authentication institutions, and its distribution is in North China, East China, South China, Southwest and Central regions, covering the plains, hills, mountain, plateau, islands with regional climate characteristics. It has a variety of geographical, climatic, and urban characteristics.

**Figure 4:**
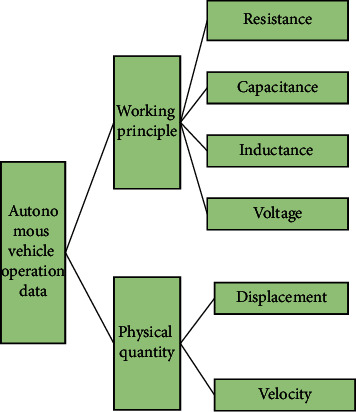
Interpretation of the data used in the experiment.

The data studied in this paper are 204 automatic driving crashes in NAIS database. In order to ensure the representativeness and typicality of the data, the scene data and the analysis method of the data were determined after the statistical analysis of 204 accident data in NAIS database, and the selected 204 cases of data are randomly selected and set.  Figure 4 shows the basic information data of the sample.

### 4.2. Experimental Results Analysis

This paper only applies the conventional Kalman filter model to the ethical design of autonomous driving crash algorithms and has achieved very good results. However, since the purpose of this paper is to prove the effectiveness of Kalman filter model in ethical design of autonomous driving crash, no other comparison algorithm is designed in this paper. In general, the larger the number of clusters, the higher the profile coefficient. The higher number of clusters may be overfitting, while the lower number of clusters may be underfitting. According to the idea of the compromise between cluster size and cluster effectiveness, the best number of families (*K*) is selected, and the best number of clusters is selected. The optimal number of clusters was determined by combining the ASW value and the minimum number of cluster samples with different cluster numbers. The rules for determining the optimal number of families are as follows: First, the proportion of the sample number of the minimum family in the total sample must be greater than or equal to a certain proportion, which is determined according to the ASW value and its changes. The second is to select the cluster number with higher ASW value under the condition of higher minimum cluster sample number. Third, when the value of ASW is high, the number of clusters with less negative contour coefficient is selected to reduce the possibility of incorrect clustering.

**Figure 5:**
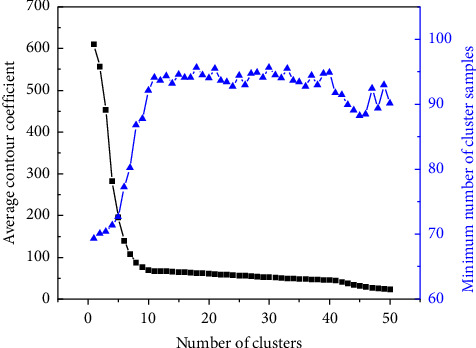
ACC values and minimum cluster samples of different cluster numbers in driving sections.

The ACC value and minimum cluster sample number under different cluster numbers of driving sections are shown in Figure 5. For the number of clusters with 912 or more, the minimum number of samples in each cluster was reduced to less than or equal to 63 (8.1% of the total sample). Because the sample size was too small and the ACC value of 10 to 20 clusters was high, i.e., above 500, the selection was not performed. In the figure, the number of 11 clusters, the number of 12 clusters, and the number of 813 clusters have high ACC values and the same minimum cluster sample number, whose ACC values are 55, 52, and 56, respectively.

**Figure 6:**
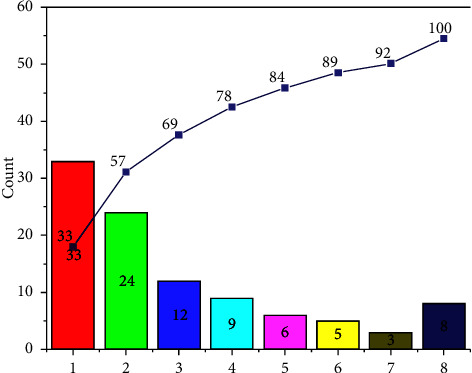
The contour coefficient of eight clusters of driving sections.

By comparing the contour coefficient graph of 6, 7, and 8 clusters (as shown in Figure 6), it can be seen that the number of samples with negative contour coefficient in the contour coefficient graph of eight clusters is significantly less; that is, fewer samples are placed in the wrong cluster, so a total of six clusters are selected as the optimal number of clusters.

**Figure 7:**
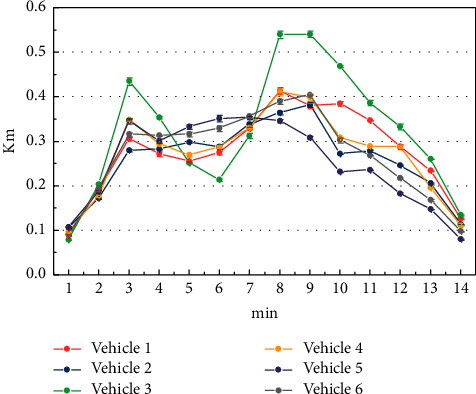
Longitudinal position change process of all vehicles in multilane simulation experiment.

Figure 7 shows the longitudinal position change process of all vehicles in the multilane simulation experiment. Because the remaining traffic time is not enough, all vehicles cannot cross the stop line at the intersection, so they need to choose the appropriate lane according to the lane selection rules and start-stop strategy to slow down and wait in line. Vehicles 1 and 3 only need to keep their original lanes and implement the start-stop strategy at the appropriate moment. For vehicle 2, since there is no vehicle ahead in the left lane, it should change lane to the left lane instead of following vehicle 1 and staying in the middle lane. At this point, each lane has been selected by a car. For vehicle 4, all three lanes can be selected at this time, but in order to avoid unnecessary movements and operations in the process of driving, it should stay in the original lane. Vehicle 5 has two options, lane change left or right. Following the traffic advice to drive on the left, the driver chooses to switch to the left lane. So far, vehicle 6 can only choose to change lane to the right to ensure that it takes the least time to pass the intersection.

**Figure 8:**
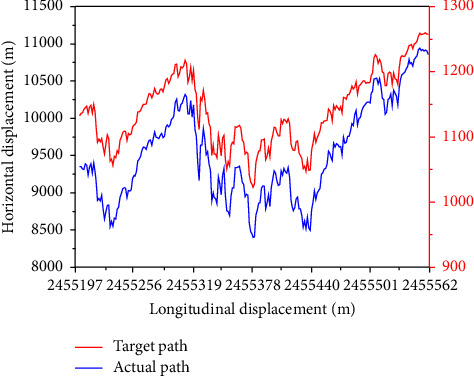
Autopilot collision risk path tracking results.

Furthermore, Figure 8 shows the autopilot collision risk path tracking results, where the red line is the target path and the blue line is the actual path. As can be seen from the figure, with the increase of the automatic driving process, both lateral and longitudinal displacements changed to different degrees. However, all the proposed machine learning algorithms can fit the real automatic driving route to a good extent. It shows that the designed algorithm not only achieves a good impact effect in the automatic driving test but also meets the requirements of algorithm design ethics. Because the Kalman filter model is only applied to the ethical design of autonomous driving crash algorithms, the characteristics of autonomous driving are quite different from those in nonautonomous driving scenario. Therefore, whether this method is applicable to nonautonomous driving scenario needs to be verified by subsequent studies.

## 5. Conclusions

Since the algorithms have become increasingly indispensable, their ability to deal with real-world problems has increased. In fact, the algorithm is an extremely rational choice in human decision-making process; that is, it does not involve subjective feelings in specific problems but makes decisions and judgments through a series of presuppositions. Human beings, due to their own limitations, often make mistakes because of irrationality, while algorithms can implement absolute rationality with strict discipline. The development of algorithms is a new stage for people to understand the unknown world, can promote people to understand themselves and summarize the experience and lessons of decision-making process, and deepen the understanding of humanity and rationality.

Vehicle safety has always been a key link of the automatic driving technology; in order to improve the safety of vehicles in the process of driving the car, a number of active safety technology appeared in the market with the collision avoidance system; the industry is more into collision warning or emergency braking its way for the realization of collision avoidance and the collision avoidance method in some special or more emergency scenarios, where an unavoidable collision can occur. In this paper, based on the Kalman filter algorithm, the algorithm ethics of automatic driving collision accident is designed and applied, and a better simulation experiment structure and algorithm ethics requirements are obtained. Although the proposed method achieves good results in ethical design of autonomous driving crash algorithms, in the future research, the Kalman filter combined with deeper structure algorithm and the application of big data scenarios are worth studying.

## Data Availability

The datasets used during the current study are available from the corresponding author on reasonable request.
